# ABDOMINAL MYOSTEATOSIS AND COGNITIVE FUNCTION: INSIGHTS FROM CARDIA STUDY

**DOI:** 10.1093/geroni/igae098.0794

**Published:** 2024-12-31

**Authors:** Adrianna Acevedo-Fontanez, Caterina Rosano, Kristine Yaffe, J Jeffrey Carr, James Greg Terry, Sangeeta Nair, Ryan Cvejkus, Iva Miljkovic

**Affiliations:** University of Pittsburgh, Pittsburgh, Pennsylvania, United States; University of Pittsburgh, Pittsburgh, Pennsylvania, United States; University of California San Francisco, San Francisco, California, United States; Vanderbilt University, Nashville, Tennessee, United States; Vanderbilt University Health Sciences, Nashville, Tennessee, United States; Vanderbilt University, Nashville, Tennessee, United States; University of Pittsburgh, Pittsburgh, Pennsylvania, United States; University of Pittsburgh, Pittsburgh, Pennsylvania, United States

## Abstract

The role of myosteatosis in cognitive decline is largely from cross-sectional studies in White populations. Longitudinal studies including Black populations, who have a higher risk of both myosteatosis and dementia than Whites, are warranted. We tested the association between abdominal myosteatosis with 5-year change in cognition in 2,501 White and Black American men (n=1,078; 42% Black) and women (n=1,423; 49% Black) from the Coronary Artery Risk Development in Young Adults (CARDIA), and whether associations vary after adjustment for demographics, center, education, muscle area, follow-up-time, baseline DSST score, smoking, physical activity, diabetes, hypertension, lipid-lowering medication, and BMI. IMAT was log-transformed, and results were standardized with effects shown per standard deviation (SD). Higher IMAT predicted a significant DSST decline in Whites (5-year decrease in DSST: b=-0.77 points for 1 SD of log IMAT, p-value=<0.01) but not in Blacks (b=-0.03, p-value=0.910). By race, higher IMAT was associated with a significant decline in DSST score. We did not see a significant difference in a 5-year decrease in DSST by sex. Abdominal myosteatosis may be a novel risk factor for cognitive decline, especially in Whites. Future studies using an expanded battery of cognitive tests, longer follow-ups, and in other populations, as well as mechanistic studies, are warranted to further confirm and advance our understanding of the novel role of myosteatosis in dementia risk.

